# Wide pH, Adaptable High Internal Phase Pickering Emulsion Stabilized by a Crude Polysaccharide from *Thesium chinense* Turcz.

**DOI:** 10.3390/molecules29184312

**Published:** 2024-09-11

**Authors:** Borong Ling, Lijun Shao, Huicong Jiang, Shufang Wu

**Affiliations:** 1 College of Light Industry and Food Engineering, Nanjing Forestry University, Nanjing 210037, China; lingbr@njfu.edu.cn (B.L.); lijunshao@njfu.edu.cn (L.S.); jhcong@njfu.edu.cn (H.J.); 2 Jiangsu Co-Innovation Center of Efficient Processing and Utilization of Forest Resources, Nanjing Forestry University, Nanjing 210037, China

**Keywords:** Pickering emulsions, *Thesium chinense* Turcz., polysaccharide, ultrasonic-assisted extraction

## Abstract

The ultrasound-assisted extraction conditions of *Thesium chinense* Turcz. crude polysaccharide (TTP) were optimized, and a TTP sample with a yield of 11.9% was obtained. TTP demonstrated the ability to stabilize high-internal-phase oil-in-water emulsions with an oil phase volume reaching up to 80%. Additionally, the emulsions stabilized by TTP were examined across different pH levels, ionic strengths, and temperatures. The results indicated that the emulsions stabilized by TTP exhibited stability over a wide pH range of 1–11. The emulsion remained stable under ionic strengths of 0–500 mM and temperatures of 4–55 °C. The microstructure of the emulsions was observed using confocal laser scanning microscopy, and the stabilization mechanism of the emulsion was hypothesized. Soluble polysaccharides formed a network structure in the continuous phase, and the insoluble polysaccharides dispersed in the continuous phase, acting as a bridge structure, which worked together to prevent oil droplet aggregation. This research was significant for developing a new food-grade emulsifier with a wide pH range of applicability.

## 1. Introduction

Pickering emulsions are mixtures of water-in-oil or oil-in-water stabilized by the adsorption of solid or colloidal particles at the interface between the two phases [1]. Due to the irreversible physical barrier formed by solid particles at the oil-water interface, Pickering emulsions can effectively resist droplet aggregation, flocculation, and Ostwald ripening, which are caused by interfacial and droplet contact interactions [2]. Pickering emulsions are widely applied in various fields such as food [3], agriculture [4], cosmetics [4], and medicine [5]. The concept of “high-internal-phase emulsions (HIPEs)”, which refers to an emulsion with a volume fraction of the dispersed phase exceeding 74%, was first proposed in the 1960s [6]. In HIPEs, droplets are tightly packed and deformed, exhibiting gel-like rheological properties [7]. This makes HIPEs significant in the food industry for replacing partially hydrogenated oils to provide a semi-solid texture or encapsulate fat-soluble actives [8].

Traditional Pickering emulsions primarily use inorganic particles, synthetic particles, or polymer particles as emulsifiers, such as SiO_2_ [9], metal oxides [10], and polystyrene [11]. Although certain types of inorganic particles are approved for use in food within specified limits (such as TiO_2_ and SiO_2_), they are often avoided in food applications due to the increasing consumer preference for green products. Additionally, some emulsifiers may harm human health; for instance, carbon nanotubes can induce fibrogenic reactions and granuloma formation [12]. Therefore, developing more natural, safer, and environmentally friendly emulsifiers can meet the growing consumer demands for Pickering emulsions in commercial food products.

Proteins are considered excellent food-grade emulsifiers due to their superior surface activity and rapid interfacial adsorption kinetics [13]. HIPEs containing protein stabilizers exhibit favorable viscoelasticity and plasticity [14]. In addition, natural polysaccharides, such as chitosan, starch, and nano-cellulose, are commonly used to stabilize Pickering emulsions. However, these two types of emulsifiers also have limitations. Proteins adsorbed at the interface unfold due to their structural constraints, leading to lateral attractive interactions and even denaturation and aggregation, which can result in decreased emulsion stability [15]. Due to high hydrophilicity, polysaccharides are difficult to wet with oil, resulting in poor emulsifying performance [16]. Modification is an effective method to improve the emulsifying performance of polysaccharides. In the case of starch, reducing the size of starch particles results in smaller Pickering emulsion droplet sizes, and the stability of the emulsion increases accordingly. Starch nanoparticles and crystals can be prepared using acid hydrolysis, enzymatic debranching and recrystallization, high-pressure homogenization, and ultrasonication [17]. However, these modification methods increase energy consumption, and chemically modified starch particles may not appeal to consumers in the growing clean-label ingredients market. Using polysaccharide–protein complex particles, such as soy protein isolate–chitosan composite nanoparticles [18] and Quinoa protein–polysaccharide electrostatic complexes [19], which are complexes of fiber polysaccharide–protein extracted from *Haematococcus pluvialis* [20], to stabilize Pickering emulsions is an effective approach to address these issues. However, most natural polysaccharide–protein complexes require processing before being used as emulsifiers (e.g., Maillard reactions [16]), making the emulsion fabrication process too complex. Therefore, it is highly important to find a readily available natural emulsifier.

*Thesium chinense* Turcz. is a traditional Chinese herb with a long history in China and Southeast Asia. It contains flavonoids, polysaccharides, alkaloids, and other nutrients. Polysaccharides are often discarded during medicine processing, despite reports indicating the antioxidant activity and application potential of *Thesium chinense* Turcz. polysaccharides [21]. In recent years, the research on *Thesium chinense* Turcz. polysaccharides has been increasing. In a previous study, we found the polysaccharide extracted from *Thesium chinense* Turcz. has a higher protein content. In this study, the crude polysaccharide with proteins was extracted from *Thesium chinense* Turcz. by ultrasonic-assisted extraction, and the ability of the crude polysaccharides to stabilize oil-water emulsions was investigated. This work will provide a basis for improving the utilization of *Thesium chinense* Turcz. and promoting the development of green and sustainable emulsifiers.

## 2. Results and Discussion

### 2.1. Optimization of the TTP Extractive Conditions

**Figure 1:**
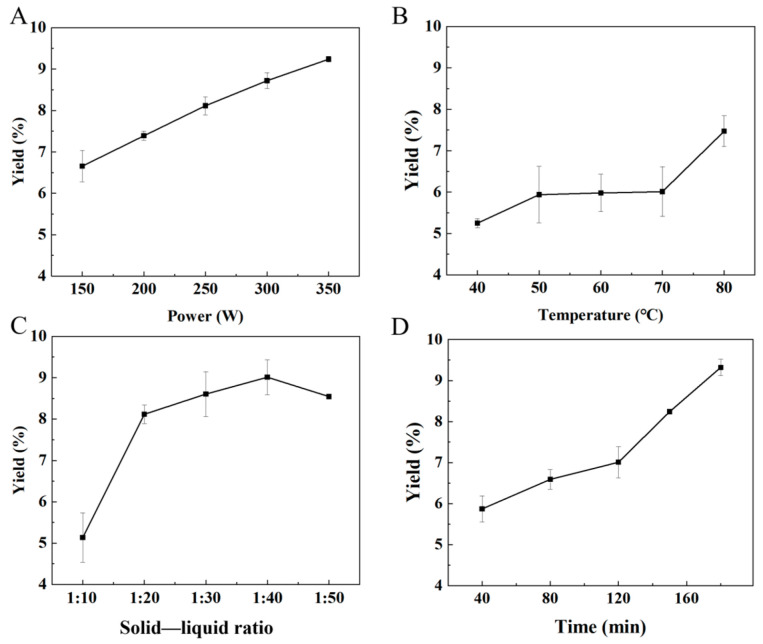
The effect of ultrasonic power (**A**), temperature (**B**), solid–liquid ratio (**C**), and time (**D**) on extraction yield.

TTPs were extracted by ultrasonic-assisted extraction. To determine the level of in-dividual factors in orthogonal experiments, a single-factor experiment was performed. The effects of extractive conditions, including temperature, time, power, and solid (g)–liquid (mL) ratio, on the yield of TTP extraction were evaluated. The results are shown in Figure 1.

Figure 1A shows that the yield of TTP increased continuously with the increase of ultrasonic power. The powerful ultrasonic energy was concentrated in the container, promoting the release of polysaccharides from the cell wall [22]. Within the set range of factors, the TTP yield increased with temperature, ultrasonic power, and extraction time. Notably, the yield reached a maximum value of 8.5% at a solid–liquid ratio of 1:40 and no longer increased. An appropriate solid–liquid ratio not only enlarged the contact between the two phases but also reduced the system’s viscosity, thereby promoting ultrasonic cavitation and facilitating TTP dissolution [23]. On the contrary, the too high solid–liquid ratio led to the dispersion of ultrasonic energy, thereby reducing the ultrasonic cavitation effect, which hindered the dissolution of polysaccharides [24].

Based on the results of the single-factor experiment, an orthogonal experiment was designed and conducted and listed in Table 1. The effect of each factor on the yield of TTP in descending order was temperature, power, time, and solid–liquid ratio. Based on this, the optimal extractive conditions were determined as a temperature of 80 °C, ultrasonic power of 350 W, extractive time of 180 min, and a solid–liquid ratio of 1:40. Validation experiments confirmed that the yield of the TTP obtained under these optimal conditions reached 11.9%. A TTP sample extracted under these optimal conditions was used for all subsequent experiments.

### 2.2. Characterization of TTP

**Figure 2:**
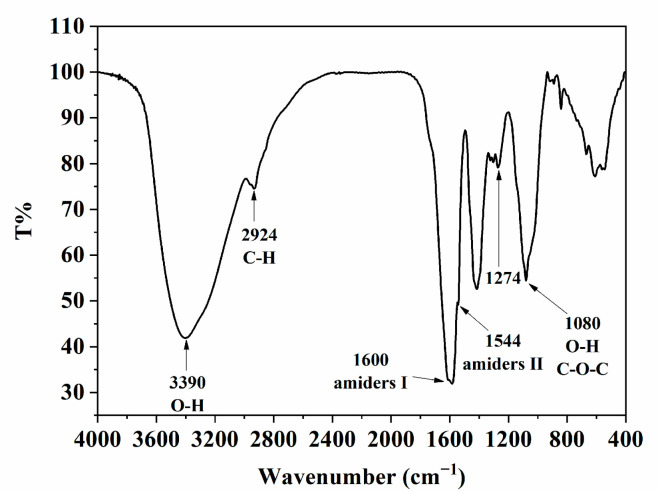
The FTIR spectrum of the TTP sample.

Through measurements, it was found that TTP contained up to 22.5% protein in addition to 45.6% carbohydrates. The solubility of TTP was determined to be 75.1 ± 0.6%. As shown in Figure 2, the broad peaks observed around 3390 cm^−1^ and 2924 cm^−1^ were attributed to the stretching vibrations of O-H and C-H (-CH_3_ or -CH_2_) in the molecules, respectively [25]. The peak near 1080 cm^−1^ corresponded to the stretching vibration of the O-H side chain and the vibration of the glycosidic bond (C-O-C) [26,27]. These are all characteristic peaks typical of carbohydrates. Peaks were observed near 1600 cm^−1^ and 1544 cm^−1^, corresponding to amide I and amide II, respectively, indicating the presence of proteins [25]. It was also suggested that covalent bonds form between proteins and polysaccharides through reactions involving carbonyl and amino groups [28,29]. A peak appeared at 1274 cm^−1^, attributed to a covalent bridge between the amino groups in the proteins and the carboxyl groups in the polysaccharides [30]. This suggested that proteins and polysaccharides form mixed molecular complexes.

### 2.3. Antioxidant Activity of TTP

**Figure 3:**
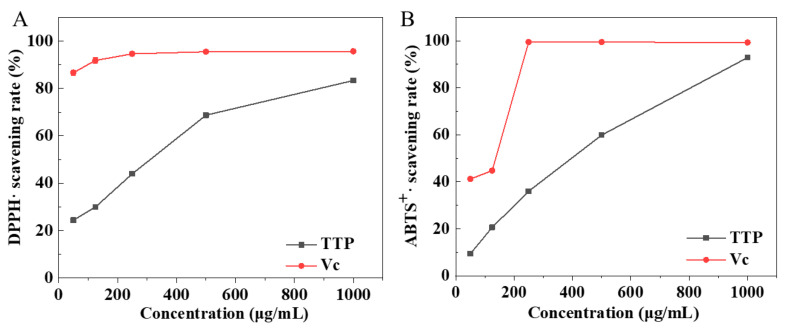
The scavenging rates of DPPH· (**A**) and ABTS^+^ (**B**) radicals at different concentrations of TTP in comparison to vitamin C (Vc).

To evaluate the antioxidant activity of TTP, the scavenging rates of DPPH· and ABTS^+^ were determined, respectively. As shown in Figure 3, at a concentration of 1000 µg/mL, the scavenging rates of TTP on DPPH· and ABTS^+^ reached 83.3% and 92.9%, respectively, which were close to those of Vitamin C (Vc) at the same concentration (95.9% and 99.2%). The excellent antioxidant performance of TTP was mainly attributed to the polysaccharides and proteins it contained, which have been confirmed to possess antioxidant properties in previous reports [31].

### 2.4. Preparation and Characteristics of Emulsions Stabilized by TTP

**Figure 4:**
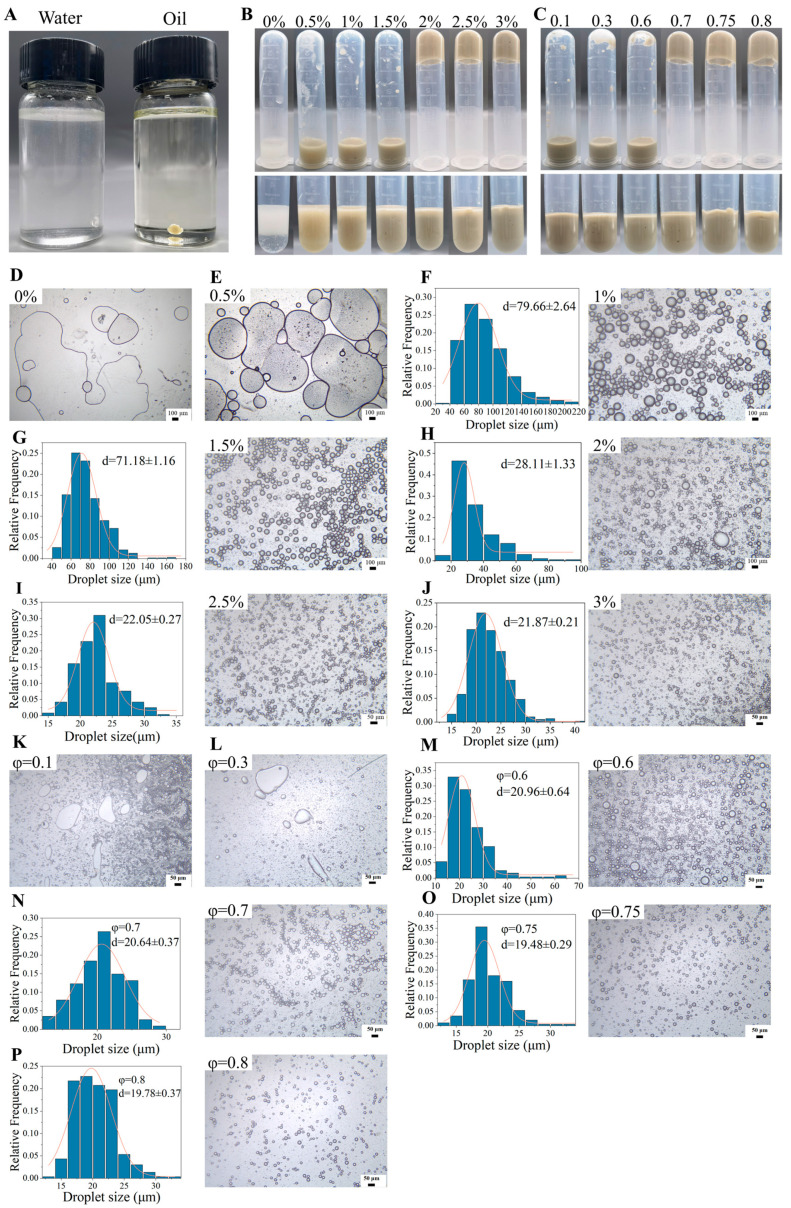
The drop test of Pickering emulsions stabilized by TTP (**A**). Visual observation of Pickering emulsions with different TTP concentrations (**B**) and with varied oil phase fractions (**C**). Particle size distribution diagram and microscopic images of Pickering emulsions with different TTP concentrations (**D**–**J**) and oil volume fractions (**K**–**P**).

A drop test determined the emulsion type as shown in Figure 4A. The emulsion drops were aggregated into a droplet in the oil but homo-dispersed in water, indicating that the emulsion stabilized by TTP was an oil-in-water emulsion [18,32].

It can be seen from Figure 4B–P that the visual and microscopic characteristics of Pickering emulsions varied with TTP content and volume fractions of oil (φ). The Pickering emulsions were well fabricated without obvious delamination when the TTP content was 0.5–3% (*w*/*v*). Moreover, the emulsion exhibited an inverted and non-flowing state as the TTP content increased to 2% (*w*/*v*) (Figure 4B). Additionally, the droplet size of the emulsion continuously decreased as the TTP content increased and became more uniform until 3.0% (*w*/*v*) (Figure 4F–J). Furthermore, in preliminary experiments, it was found that when the TTP content reached 3.5%, the particle size increased (as shown in Figure S1). Consequently, the Pickering emulsions with 3% (*w*/*v*) TTP content were selected for further studies. The characteristics of the emulsions with different φ values stabilized by TTP are shown in Figure 4C. Within 0.1–0.8 of φ, the Pickering emulsions did not exhibit obvious delamination. As the φ value increased to 0.7 or above, an inverted non-flowing emulsion was formed, and the emulsion became more viscous, consistent with the characteristics of the high-internal-phase emulsion. The interaction between droplets within the Pickering high-internal-phase emulsion formed a percolated network structure of emulsion gel, which could provide additional stability and prevent coalescence [33]. The droplet size of the emulsion decreased with φ value until φ = 0.8. When the φ increased to 0.85, the droplet size increased (as shown in Figure S2). A high-internal-phase Pickering emulsion (HIPE) with φ = 0.8 was selected for further study.

### 2.5. Stability of HIPEs Stabilized by TTP

Complex and varied food processing requires Pickering emulsions to be stable under different environmental stresses [34]. Hence, the influence of pH, ionic strength, and temperature on the stability of HIPEs stabilized by TTP was evaluated by observing the appearance and measuring the droplet size and viscosity.

#### 2.5.1. Effects of pH

**Figure 5:**
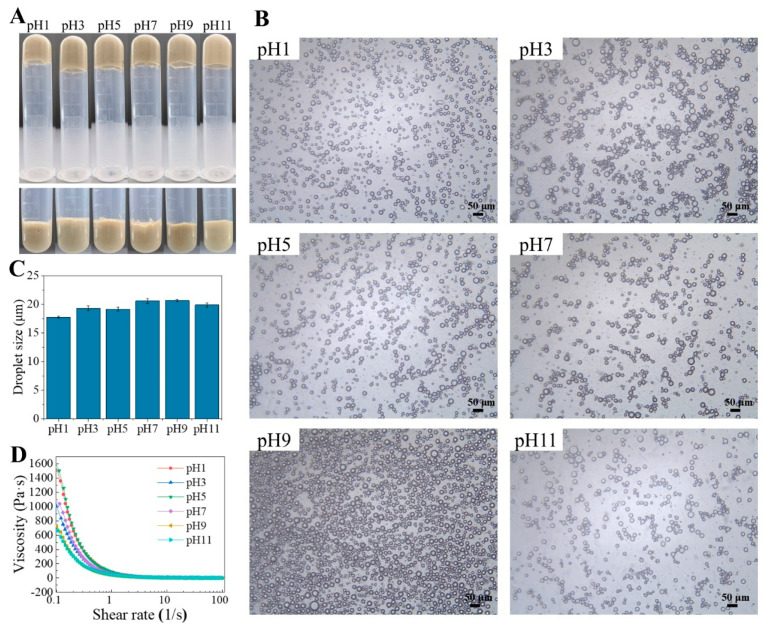
Digital photographs (**A**), microscopic images (**B**), average droplet sizes (**C**), and viscosity (**D**) of HIPEs stabilized by TTP at different pH.

As can be seen in Figure 5A, Pickering emulsions were successfully fabricated at a pH value of 1 to 11. There was no obvious stratification of the emulsions even in an extremely acidic environment (pH 1). The average size of the oil droplets was 17–21 μm, where the droplet was slightly larger at a pH of 7 to 9.

The rheological behavior is important for the stability and functionality of the Pickering emulsions [35,36]. As shown in Figure 5D, the emulsions exhibited typical shear thinning behavior (non-Newtonian fluid) as the shear rate increased from 0 to 100 s^−1^. This shear thinning reflects the disruption of the network structure in the emulsion and its gradual deformation as the shear rate increases [9]. Similar behavior was observed in Pickering emulsions stabilized by soy protein isolate–bacterial nanocellulose complexes [37]. It is worth noting that this HIPE stabilized by TTP had higher viscosity under acid conditions, implying better stability. This phenomenon has also been reported in previous studies. It was suggested that when the pH was below the isoelectric point of protein, it enhanced the electrostatic interaction between protein and polysaccharide, which was conducive to the formation of a mixed molecular complex and prevented protein precipitation. At the same time, in the continuous phase of the emulsion, the proteins formed electrostatic bridges between the polysaccharides, creating a network that increased the viscosity of the emulsion [38]. 

Many natural polysaccharide-stabilized Pickering emulsions demonstrated stability under a broad pH range. For instance, modified cellulose crystals could form stable emulsions within the pH range of 4 to 7 [39], and natural protein–polysaccharide hybrid nanoparticles extracted from Lactobacillus plantarum could form stable Pickering emulsions within the pH range of 3 to 11 [23]. The Pickering emulsions stabilized by TTP exhibited excellent pH stability, maintaining stability across a broad pH value of 1 to 11, offering a wide range of applications.

#### 2.5.2. Effects of Ionic Strengths

**Figure 6:**
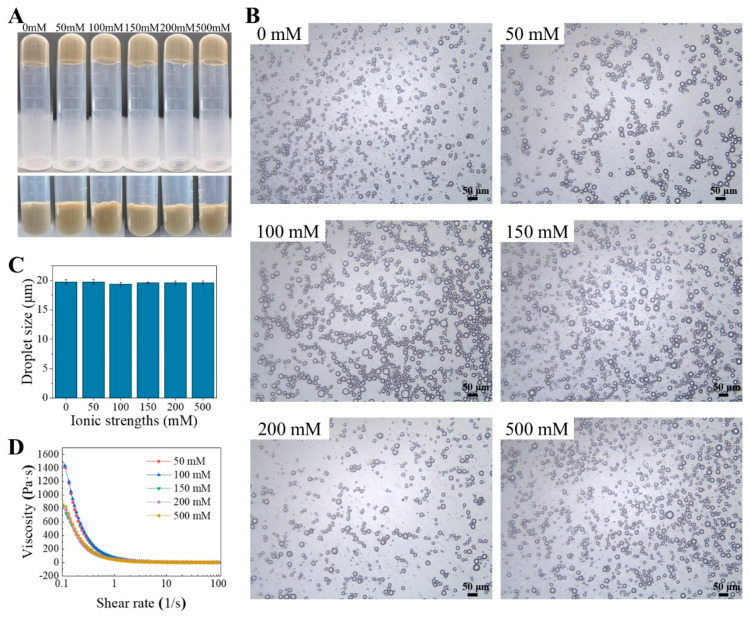
HIPEs stabilized by TTP at different ionic strengths. (**A**) Digital photographs, (**B**) microscopic images, (**C**) the average droplet, (**D**) and viscosity curves.

Salt not only reduces the surface potential of colloidal particles but may also lead to flocculation and precipitation of the particles. Therefore, it is necessary to study the effect of ionic strength on the Pickering emulsions stabilized by TTP. As can be seen from Figure 6C, there was no significant difference in the visual appearance and size of the emulsions prepared at ionic strength concentrations ranging from 0 to 500 mM. However, the viscosity of the emulsion was higher at low ionic concentrations (Figure 6D). This was probably because the addition of salt ions shielded the electron layer on the surface of the droplets, and the electrostatic repulsion between the droplets was weakened, resulting in slight flocculation of droplets of the emulsion [40,41]. Overall, the HIPEs stabilized by TTP exhibited excellent salt stability.

#### 2.5.3. Effects of Temperature

**Figure 7:**
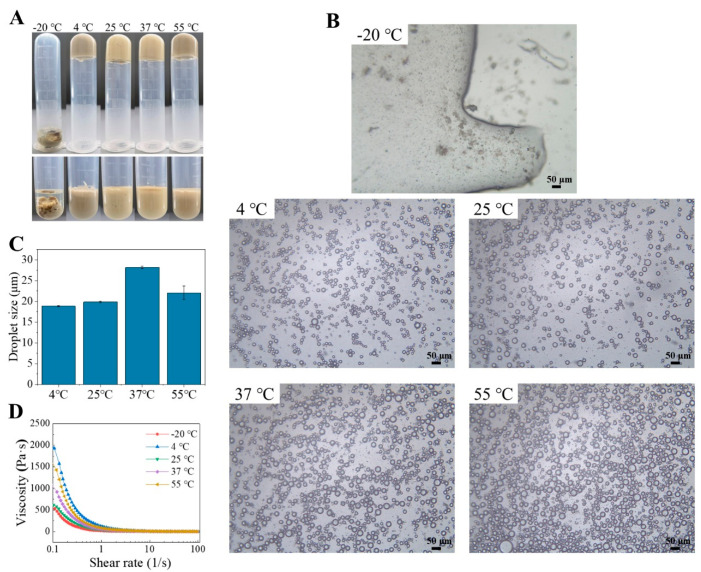
Digital photographs (**A**), microscopic images (**B**), average droplet sizes (**C**), and viscosity curves (**D**) of HIPEs stabilized by TTP at different temperatures.

The HIPEs stabilized by TTP were stored at various temperatures of −20 °C, 4 °C, 25 °C, 37 °C, and 55 °C for three days to evaluate their storage stability. The appearance photographs of the emulsion showed that the emulsions stored at −20 °C lost their original equilibrium and appeared demulsified, while the oil droplets remained relatively homogeneous after storage at 4 °C to 55 °C for three days (Figure 7A,B). This indicated that the emulsion stabilized by TTP is adaptable to most processing and storage temperatures.

### 2.6. Microstructure of HIPEs Stabilized by TTP

**Figure 8:**
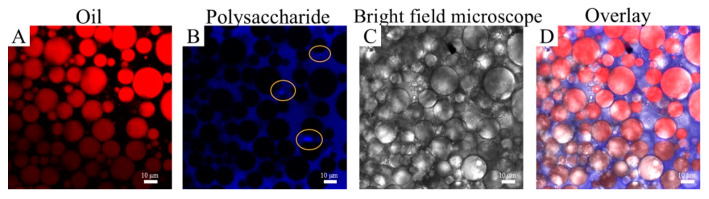
CLSM images of the Pickering emulsions stabilized by TTP.

**Figure 9:**
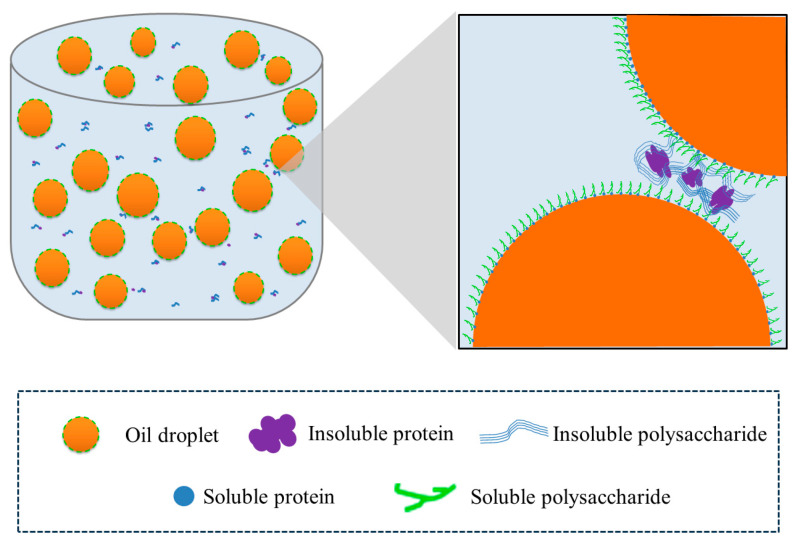
The schematic diagram of the TTP-stabilized Pickering emulsion mechanism.

To elucidate the stability mechanism of HIPEs stabilized by TTP, the microstructure of the emulsions was observed by CLSM. Oil and polysaccharides of the emulsion were stained red and blue by Nile Red and Calcofluor White, respectively, before being observed [42]. As shown in Figure 8, the TTP was present in the emulsion in two ways: one part was uniformly dispersed in the continuous phase. In contrast, the other part was mainly distributed between the droplets as particles. As mentioned above, the soluble component in TTP was relatively high at 75.1 wt%, which consisted of soluble polysaccharides and soluble proteins. It has been reported that water-soluble polymers effectively reduce the molecular forces between solvents, thereby decreasing interfacial tension [43]. Therefore, the soluble polysaccharides and proteins in TTP were adsorbed at the oil-water interface, reducing interfacial tension and stabilizing the emulsion. Additionally, the insoluble polysaccharides and proteins in TTP formed a bridging structure that prevented the aggregation of the oil droplets, thereby stabilizing the emulsion. This fits with the stabilization mechanism of Pickering emulsification [44]. In summary, the possible stabilization mechanism of emulsions stabilized by TTP was elucidated; the unique stabilizing effect of the emulsion stabilized by TTP was due to the synergistic interaction between the soluble and insoluble fractions. Figure 9 provides a schematic representation of TTP stabilization emulsion.

## 3. Materials and Methods

### 3.1. Materials and Reagents

*Thesium chinense* Turcz. was purchased from the Chinese medicine market in Bozhou, Anhui Province. Sunflower oil was purchased from the supermarket. Calcofluor White was purchased from Coolaber CO., Ltd. (Beijing, China). Nile red was purchased from Yuanye Biotechnology CO., Ltd. (Shanghai, China). All other chemicals used in this study were of analytical grade.

### 3.2. Pretreatment of Raw Materials

To remove certain components from *Thesium chinense* Turcz., soaking in solvents could yield better subsequent processing results. For instance, to eliminate monosaccharides, oligosaccharides, and some alcohol-soluble pigments, soak the powder in 95% ethanol at 4 °C for 12 h. To remove certain flavonoids and pigments, soak it in petroleum ether at 4 °C for 12 h. After these steps, dry the obtained powder at 50 °C. Sieve the dried powder through an 80-mesh sieve to obtain the fine powder, which can be stored for future use.

### 3.3. Ultrasound-Assisted Extraction of TTP

TTP was extracted using an ultrasonic device (SB-5200DTD, Scientz, Ningbo, China) under the set solid–liquid ratio, temperature, ultrasonic time, and ultrasonic power. After ultrasonic-assisted extraction, the liquid was filtered, and a threefold volume of 95% ethanol solution was added to the filtrate. The mixture was then precipitated at 4 °C for 12 h, and the precipitate was freeze-dried to obtain TTP powder. The extraction yield was defined as the percentage of the total solid mass of total polysaccharides and proteins to the mass of dried powder of pre-treated *Thesium chinense* Turcz.

### 3.4. Chemical Composition and Water Solubility of TTP Analysis

The total protein content of TTP was determined using the BCA protein concentration assay kit (Coolaber, Beijing, China). The phenol-sulfuric acid method was used to determine the total carbohydrate content, using glucose as the standard.

We accurately weighed 0.2 g of TTP powder and mixed it with 10 mL of deionized water, then stirred magnetically at 30 °C for 30 min. The mixture was then centrifuged at 7000 rad/min for 10 min. The supernatant was collected and transferred to a weighing bottle, then dried at 105 °C to a constant weight. The water solubility was calculated as the percentage of the mass of the dried soluble substance to the initial dry mass of the TTP.

### 3.5. FTIR Spectra of TTP Analysis

The infrared analysis of TTP samples was carried out using the KBr tablet pressing method. Firstly, 1.00 mg of the sample was accurately weighed and ground with 100 mg of KBr. Subsequently, the mixture was pressed into a tablet. The analysis was performed using a VERTEX 80V device from Bruker, Karlsruhe, Germany. The data collection range spanned from 4000 to 400 cm^−1^. 

### 3.6. Antioxidant Activity of TTP Assay

The DPPH radical scavenging ability of the TTP sample was assessed using a published method [45]. Dispersions of TTP at different concentrations (50, 120, 250, 500 and 1000 μg/mL) were added to centrifuge tubes. Subsequently, 2 mL of DPPH solution (0.1 mM) was added, mixed, and incubated in the dark for 30 min. The absorbance of the reaction mixture was measured at 517 nm. The DPPH radical scavenging rate was calculated using Equation (1):

(1) $$\mathrm{Scavenging}\;\mathrm{effect}\;\mathrm{of}\;DPPH\cdot \left(\%\right)=\frac{A_{0}-(A_{1}-A_{2})}{A_{0}}\times 100$$

where A_1_ was the absorbance of the sample, A_2_ was the absorbance of the solvent control (alcohol instead of DPPH), and A_0_ was the absorbance of the blank (water instead of the sample).

The ABTS radical scavenging capacity of TTP samples was evaluated according to established methods [46]. The ABTS solution (7.4 mM) and potassium persulfate solution (2.6 mM) were mixed in equal volumes and kept in the dark at room temperature for 16 h. Then, the solution was diluted until the absorbance value of 0.70 (±0.02) at 734 nm. Next, 0.5 mL of TTP suspension (50, 125, 250, 500, and 1000 μg/mL) was mixed evenly with 2.5 mL of ABTS solution. The absorbance was measured at 734 nm after reacting at room temperature for 6 min. The ABTS radical scavenging rate was calculated using Equation (2):

(2) $$\mathrm{Scavenging}\;\mathrm{effect}\;\mathrm{of}\;{\mathrm{ABTS}}^{+}\left(\%\right)=\frac{A_{0}-(A_{1}-A_{2})}{A_{0}}\times 100$$

where A_1_ was the absorbance of the sample, A_2_ was the absorbance of the solvent control (water instead of ABTS^+^), and A_0_ was the absorbance of the blank (water instead of the sample).

### 3.7. Preparation of Pickering Emulsions Stabilized by TTP

The Pickering emulsion was prepared with slight modifications to the method of Zeng et al. [33]. For the optimization of TTP concentration, 0, 10, 20, 30, 40, 50, and 60 mg of TTP were precisely weighed in separate tubes, then 0.6 mL of water and 1.4 mL of oil were added to the tubes to make the final TTP concentrations at 0, 0.5, 1, 1.5, 2, 2.5 and 3% (*w*/*v*). After vortex mixing for 45 s, the mixture was sheared by a homogenizer (XHF-DY, Scientz, Ningbo, China) at 13,500 rpm for 2 min, followed by a 30 s interval, and then homogenized again for another 2 min.

To optimize the oil–water ratio, the volume fractions of oil (φ) were set to 0.1, 0.3, 0.6, 0.7, 0.75, and 0.8, and the final concentration of TTP in Pickering emulsions was set at 3% (*w*/*v*). After vortex mixing, the mixture was homogenized at room temperature (13,500 rpm, 2 min, twice). After standing for 1 h, the appearance of the emulsion was photographed. The microstructure of the emulsion was observed under a microscope, and the average droplet size was measured from the microscopic images. This allowed the determination of the optimal TTP concentration and oil–water ratio.

### 3.8. Characterization of the Pickering Emulsions Stabilized by TTP

The type of Pickering emulsion was determined according to the method of Yang et al. [18]. The specific procedure was as follows: the emulsion was added to a bottle of water or peanut oil. If the emulsion dispersed rapidly in peanut oil but aggregated in pure water, it was considered a water-in-oil type emulsion; otherwise, it was an oil-in-water type emulsion [9]. 

The microscopic morphology of the emulsions was observed using an optical microscope. A drop (about 15 µL) of the emulsion stabilized by TTP was added to the slide, dispersed with a small amount (about 50 µL) of distilled water, and then observed and photographed at 100× magnification. Using Nano Measurer 1.2 software [47], 100 droplets in the microscope images were selected and labeled. The data obtained were used to create a particle size distribution graph using Origin 2021 software.

The rheological properties of the Pickering emulsions stabilized by TTP were evaluated using a rheometer MARS60 (Thermo Fisher Scientific, Dreieich, Germany). Measurements were conducted at 25 °C with a shear rate range of 0.01 to 100 s^−1^. The viscosity of the emulsion was recorded as a function of the shear rate [48].

The microstructure of the emulsion was analyzed using a confocal laser scanning microscope (CLSM). The oil and polysaccharides of the emulsions were stained with Nile red (0.1 wt%, in isopropanol) and Calcofluor White (0.1 wt%, in dimethyl sulfoxide), respectively [23]. The excitation wavelengths of Nile Red and Calcofluor White were 488 nm and 405 nm, and the fluorescence emission was detected at the wavelengths of 539 nm and 435 nm, respectively.

### 3.9. Stability of Pickering Emulsions Stabilized by TTP

Pickering emulsions under different environmental stresses were prepared with slight modifications based on previous study [18]. TTP (3%, *w*/*v*) was dispersed in aqueous solutions with different pH values (1, 3, 5, 7, 9, 11) or ionic strengths (0, 50, 100, 150, 200, 500 mM). Then, the Pickering emulsion (φ = 0.8) was prepared according to the method in Section 3.7, and the droplet size was measured after standing for 1 h. To investigate the effect of temperature, Pickering emulsions stabilized by TTP (3%, *w*/*v*, φ = 0.8) were stored at different temperatures (−20, 4, 25, 37, 55 °C) for 3 days. Subsequently, the droplet sizes of the emulsions were measured.

### 3.10. Statistical Analysis

The data were expressed as the mean ± standard deviation of three replicates. The difference between the sample and the control group was determined by the software SPSS 26.0 (IBM, Armonk, NY, USA) using the least significant difference (LSD) multi-range test. *p* < 0.05 indicated significant difference, and *p* < 0.01 indicated extremely significant difference. The analysis of experimental results was performed using Origin 2021 software.

## 4. Conclusions

In this study, the ultrasound-assisted extraction conditions of TTP were optimized, and a TTP sample with 11.9% extractive yield was obtained. TTP exhibited good antioxidant capacity across a wide concentration range, and at a concentration of 1000 µg/mL, its scavenging rates for DPPH· and ABTS^+^ reached 83.3% and 92.9%, respectively. TTP exhibited excellent emulsion stabilizing ability, enabling the formation of high internal phase emulsions with volume fractions of oil up to 80%. The HIPEs stabilized by TTP demonstrated exceptional pH adaptability, remaining stable within a range of 1–11, even in highly acidic environments (pH = 1). Additionally, it is suitable for most emulsion processing and storage environments (salt ion concentrations from 0 to 500 mM, temperatures from 4 to 55 °C). It was hypothesized that the stabilization mechanism of TTP on emulsion was a synergistic effect of soluble components and insoluble components. This work provided a strategy for preparing food-grade Pickering emulsions with wide pH adaptability using crude polysaccharides from *Thesium chinense* Turcz. The TTP-stabilized HIPE has potential in high-value applications such as drug delivery, food additives, and alternatives to hydrogenated fats.

## Figures and Tables

**Table 1:** Orthogonal experimental results.

Levels	Factor				Yield (%)
	Solid–Liquid Ratio	Time (min)	Temperature (°C)	Power (W)	
1	20	120	60	250	4.74
2	20	150	70	300	8.05
3	20	180	80	350	9.24
4	30	120	60	250	6.35
5	30	150	70	300	8.19
6	30	180	80	350	7.52
7	40	120	60	250	9.77
8	40	150	70	300	6.73
9	40	180	80	350	8.10
K1	7.34	6.95	6.33	7.01	/
K2	7.35	7.66	7.50	8.4	/
K3	8.20	8.29	9.07	7.44	/
R	0.86	1.33	2.73	1.43	/

## Data Availability

The original contributions presented in the study are included in the article/Supplementary Material, further inquiries can be directed to the corresponding author.

## Supplementary Materials

The following supporting information can be downloaded at: https://www.mdpi.com/article/10.3390/molecules29184312/s1, Figure S1. Visual observation of Pickering emulsions with 3.5% TTP concentrations (A); Microscopic images (B); Particle size distribution diagram (C). Figure S2. Visual observation of Pickering emulsions with 0.85 oil phase fractions (A); Microscopic images (B); Particle size distribution diagram (C).
