# Supplementary material for: Wide pH, Adaptable High Internal Phase Pickering Emulsion Stabilized by a Crude Polysaccharide from Thesium chinense Turcz
Source: Molecules. 2024 Sep 11;29(18):4312. doi: 10.3390/molecules29184312 (PMC11434410; doi:10.3390/molecules29184312)
Supplement: Supplementary file 1 [file molecules-29-04312-s001.zip › molecules-3138177-supplementary.pdf]

Supporting Information

Wide pH, Adaptable High Internal Phase Pickering Emulsion Stabilized by a Crude Polysaccharide from *Thesium chinense* Turcz.

Borong Ling [1,†], Lijun Shao [1,2,†], Huicong Jiang [1,2] and Shufang Wu [1,2,*]

[1] College of Light Industry and Food Engineering, Nanjing Forestry University, Nanjing 210037, China
[2] Jiangsu Co-Innovation Center of Efficient Processing and Utilization of Forest Resources, Nanjing Forestry University, Nanjing 210037, China
* Correspondence: shufangwu@njfu.edu.cn
† These authors contributed equally to this work.

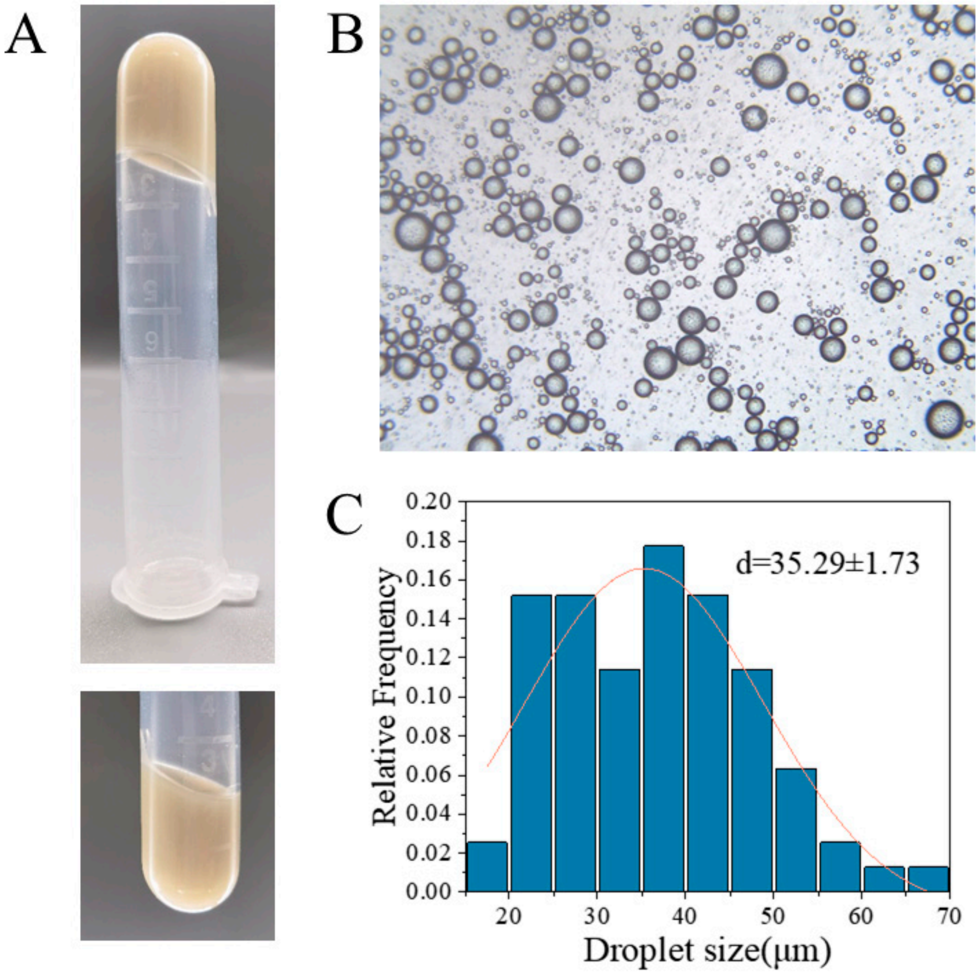

Figure. S1 Visual observation of Pickering emulsions with 3.5% TTP concentrations (A); Microscopic images (B); Particle size distribution diagram(C).

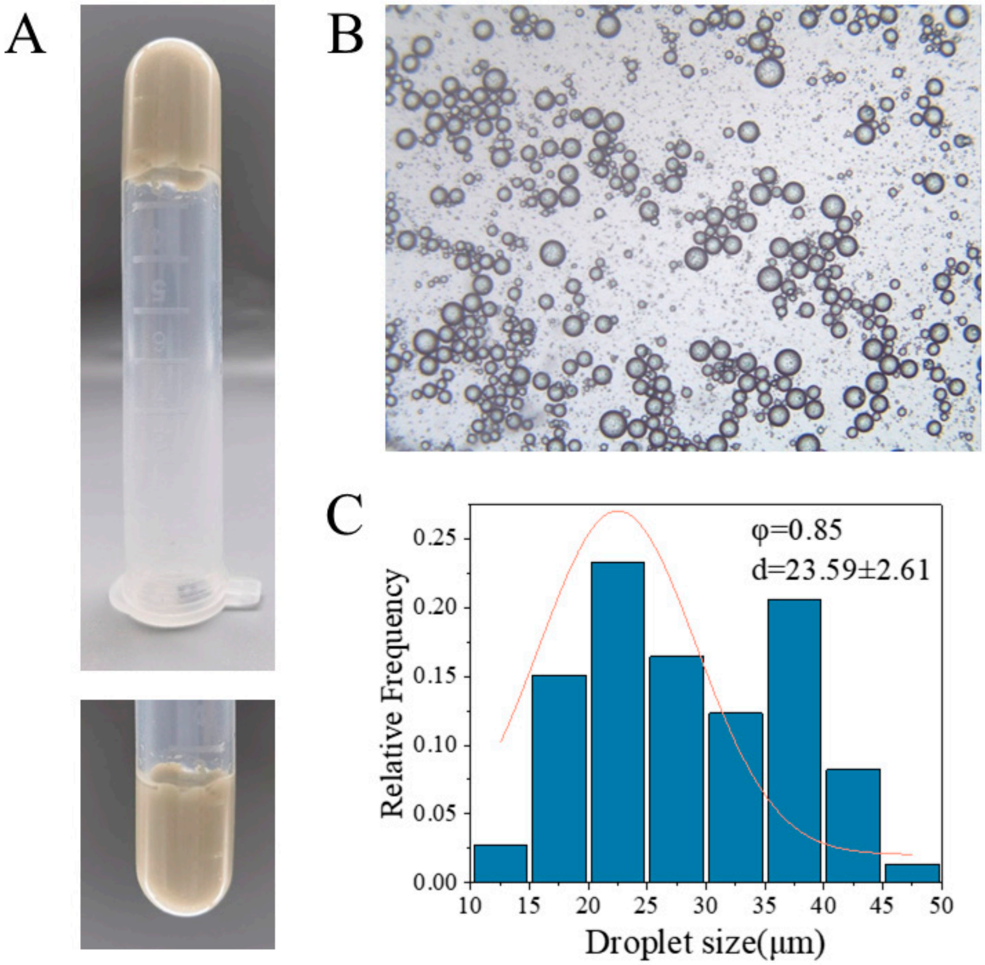

Figure. S2 Visual observation of Pickering emulsions with 0.85 oil phase fractions (A); Microscopic images (B); Particle size distribution diagram(C).
